# Long-Term Adherence to Onabotulinum Toxin-A Intradetrusor Injections for Neurogenic Dysfunction in Children—A Retrospective Single-Center Evaluation

**DOI:** 10.3390/toxins16070303

**Published:** 2024-07-01

**Authors:** Chiara Pellegrino, Valentina Forlini, Maria Luisa Capitanucci, Gessica Della Bella, Giovanni Mosiello

**Affiliations:** 1 Division of Neuro-Urology, Bambino Gesù Children’s Hospital, IRCCS, Piazza di Sant’Onofrio, 4, 00165 ERN eUROGEN Affiliated Center, 00118 Rome, Italy; valeforlini01@gmail.com (V.F.); mluisa.capitanucci@opbg.net (M.L.C.); mosiello@opbg.net (G.M.); 2 Pediatric Surgery Division, University of Genova, via Balbi 5, 16126 Genoa, Italy; 3 Neurorehabilitation and Adapted Physical Activity Day Hospital, Bambino Gesù Children’s Hospital, IRCCS, Piazza di Sant’Onofrio, 4, 00165 Rome, Italy; gessica.dellabella@opbg.net

**Keywords:** botulinum toxin-A, neurogenic bladder, pediatric, neurotoxin, neurogenic lower urinary tract dysfunction, pediatric urology, spina bifida, neurogenic detrusor overactivity

## Abstract

Onabotulinum Toxin-A (BTX-A) is a second-line treatment for neurogenic bladder (NB). It requires repeated injections over time, which is a possible limit for long-term adherence, especially in children, as general anesthesia is required. Almost 50% of adults discontinue therapy; few data on pediatric patients are present. The aim of this study is to share our long-term experience of BTX-A adherence in children. This study is a retrospective review of 230 refractory NB patients treated with BTX-A. The inclusion criteria were ≥3 treatments and the first injection performed ≥10 years before the study endpoint. Fifty-four patients were included. Mean follow-up was 10.2 years; mean treatment number was 6.4 for each patient. During follow-up, 7% did not need BTX-A anymore; 76% discontinued therapy, with a prevalence of acquired NB (64% acquired vs. 34% congenital; *p* = 0.03); sex-based and urodynamic findings did not influence the discontinuation rate (*p* = 0.6, *p* = 0.2, respectively). Considering those who withdrew from the therapy, 43% were lost to follow-up/died after a mean of 7.5 years (although 33% still experienced clinical efficacy); 33% changed therapy after a mean of 5.8 years (with reduced efficacy in 22%, persistent efficacy in 11%). BTX-A is a safe and effective therapy for pediatric patients. The treatment abandonment rate is higher for children than for adults; no specific reasons were highlighted. It is necessary to evaluate any age-specific factors to explain these data.

## 1. Introduction

Intradetrusor injections of Onabotulinum Toxin-A (BTX-A) are considered a safe and effective therapy for children with neurogenic bladder and are aimed at protecting kidney function and improving continence. The first clinical data regarding the use of Onabotulinum Toxin-A in the treatment of neurogenic lower urinary tract dysfunction (NLUTD) date to 2000; it was not until 2002 that the first reports of its use in the pediatric population emerged [1,2].

The most frequent cause underlying NLUTD in the pediatric population is congenital neuronal tube defects, with an incidence of 9.1/10,000 births in Europe, especially due to “open” or “closed” spina bifida (SB). Acquired forms of neurogenic bladder (e.g., spinal cord injury, iatrogenic lesions, tumors, systemic pathologies) are less frequent. In “open” forms of SB, such as myelomeningocele, the management of bladder emptying takes place from birth, with a Clean Intermittent Catheterization (CIC) program and the possible addition of pharmacological therapy with anticholinergic drugs (e.g., oxybutynin) to avoid renal damage (reported in 50% of these patients). Over time, further clinical approaches may become necessary to control NLUTD, including treatment with BTX-A. On the other hand, “closed” forms of SB, known as occult spinal dysraphism (OSD), do not always lead to an immediately evident pathological pattern. The clinical manifestation of OSD can be subtle, resulting in a slow onset of mild urological symptoms, which can eventually damage the upper urinary tract (UUT). Veenboer et al. evaluated, in a systematic review, the survival of children with SB into adulthood, reporting a risk of death of approximately 1% per year between the ages of 5 and 30 years old. The main cause of death in these patients remains renal failure, representing 30% of all deaths. These data are extremely important, especially considering that at birth, most SB patients have no UUT anomalies, but up to 60% of them will develop UUT deterioration due to bladder dysfunction and NLUTD [3,4,5,6,7,8,9,10,11,12].

NLUTD can manifest clinically as urinary frequency and urgency, incontinence, urinary retention, recurrent urinary tract infections (UTIs), and the appearance of urinary stones. These clinical symptoms can be associated with hydronephrosis, vesicoureteral reflux (VUR), renal scars, reduced renal function, and even renal failure. All these clinical entities reflect an alteration of bladder functioning and are the characteristics of a neurogenic bladder. Urodynamic examination can make bladder dysfunction evident, with various pathological patterns, such as the overactive bladder (OAB), low-compliance bladder (LCB), or detrusor–sphincter dyssynergia. For patients maintaining spontaneous micturition despite having neurogenic bladder dysfunction, a non-invasive urodynamic test (uroflowmetry) can also be useful [1,8,12,13].

Early management of NLUTD is associated with better preservation of the upper urinary tract. In children, as in adults, CIC and anticholinergic drugs represent the first-line treatment of NLUTD. When resistance or poor tolerance of anticholinergics appears, BTX-A intradetrusor injection is one of the most popular therapeutic options, with an increasing popularity in pediatric patients over the last 25 years [3,13,14,15].

As reported in the literature, about 39.6–49.1% of adult patients discontinue treatment over time due to BTX-A’s unsatisfactory effects. Alternatively, patients request to switch to another therapy or return to anticholinergic drugs, even if with less symptom control. More than 50% of patients abandon this treatment 10 years after the first injection. Most long-term studies on adult patients evaluate a 10-year follow-up. Due to the scarcity of data reported in the literature on the long-term follow-up of BTX-A adherence in children, we decided to evaluate and share our experience in pediatrics with the hope of contributing to the analysis and understanding of the problem relating to poor adherence to BTX-A therapy [14,16,17].

## 2. Results

Among 230 children treated with BTX-A injection from 1997 to 2022 in our pediatric urology division, 54 patients met the inclusion criteria. The first patient data included in our study were collected in 2003 because patients from 1997 to 2002 were lost to follow-up and/or underwent < 3 BTX-A treatments.

Of these 230 patients, 176 were excluded: 117 children started BTX-A therapy after 2012; 41 had fewer than three treatments; 18 patients had incomplete medical records.

Of the 54 patients enrolled, 24 were male (44%) and 30 were female (56%).

We found a congenital cause of neurogenic bladder in 69% of patients, mainly caused by myelomeningocele; 31% had an acquired etiology. The causes of neurogenic bladder in our patients are specified in Table 1.

As regards urodynamic examinations before treatment, overactive bladder (OAB) was observed in 29 (54%) patients, while the remaining 25 (46%) had a high-pressure low-compliance bladder (LCB).

The mean age at first BTX-A treatment was 8.2 years (range 1.2–19.1, SD 5.8), and the mean follow-up period (defined as the time interval between first BTX-A injection—last evaluation) was 10.2 years (range 1.7–18.8, SD 3.9).

BTX-A injections were repeated approximately every 16 months (range 6–53, SD 3.9), with an average of 6.4 treatments for patients (range 3–23, SD 3.3). The mean interval between the first and the last treatment was 6.8 years (range 1.7–15.6, SD 3.8).

**Figure 1:**
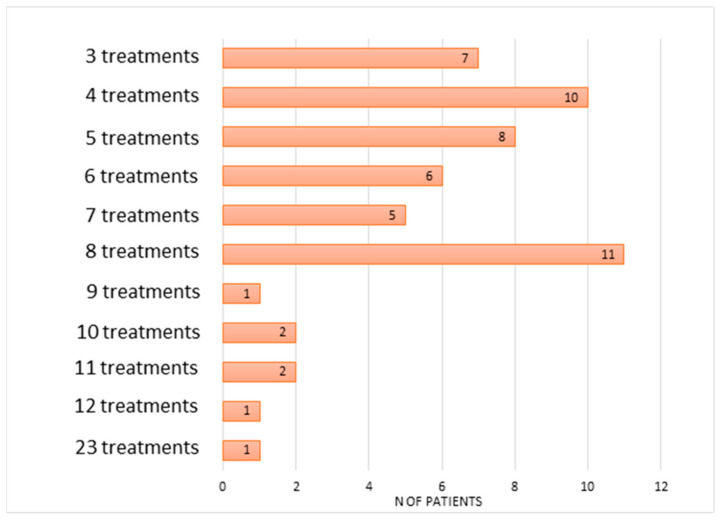
A progressive reduction in the number of patients subjected to a greater number of retreatments (therapy abandonment). N of patients: number of total patients (54).

Figure 1 shows the progressive reduction of the number of patients who underwent an increased number of retreatment.

**Figure 2:**
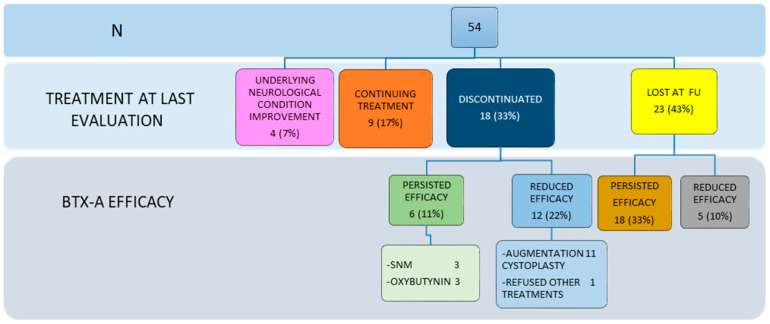
Long-term adherence and efficacy of BTX-A. FU: follow-up; SNM: sacral neuromodulation.

Long-term adherence and BTX-A efficacy are shown in Figure 2.

As shown in Figure 2, 4 patients (7%) discontinued treatment due to improvements in underlying neurological conditions, eliminating the need for further BTX-A injections (pink box): 3 of these patients saw improvement after surgical treatment for tethered cord, one patient, who had neurogenic bladder due to an arachnoid cyst, underwent surgery.

At the last available follow-up, 17% (9/54) of all patients (orange box) were still receiving regular BTX-A treatment: this group had an average of 10.5 treatments (range 8–23, SD4.8), and the mean interval between the first and the last treatments was 11.2 years (range 6.8–14.7, SD 3).

Thirty-three percent (18/54) of our cohort discontinued treatment (blue box) after an average of 5.5 injections (range 3–12, SD 2.3), and BTX-A treatment was halted after an average period of 5.8 years from the first treatment (range 2–14.1, SD 3.2). More specifically, six patients of this group (green box) chose to switch to another treatment despite the persistent efficacy of BTX-A: 3 underwent sacral neuromodulation (SNM) implantation, and 3 opted to revert to anticholinergic drugs (oxybutynin). Twelve out of 18 patients (light-blue box) discontinued treatment due to reduced efficacy of BTX-A: 11 underwent ileocystoplasty creation, with 10 also having a continent catheterizable channel created (9 had Mitrofanoff appendicovescicostomy and 1 had Monti channel); 1 patient refused further treatments. The mean duration of treatment for this subgroup of 12 patients with reduced efficacy was 5.1 years (range 2–13.8, SD 3.3), which also represents the time interval gained by delaying major surgery through BTX-A treatment.

Nearly half of our patients (43%, 23/54) were lost to follow-up after receiving an average of 5.7 treatments (range: 3–10, SD 2) and a mean follow-up of 7.5 years (range: 1.7–13.7, SD 3.3). Eighteen patients (brown box) were still responding at the last evaluation and had the next BTX-A treatment scheduled (but not administered); the other 5 patients (grey box) were lost to follow-up after experiencing secondary failure of BTX-A and refusing other alternative treatments.

If we exclude from our analysis 4 patients who no longer required BTX-A treatment (pink box), 17/50 (34%) experienced reduced BTX-A efficacy over time (light-blue and grey boxes in Figure 2), while BTX-A long-term efficacy was recorded in 33/50 (66%) patients (orange, green and brown boxes in Figure 2).

In conclusion, 76% of our patients discontinued BTX-A therapy during our follow-up period (blue and yellow boxes). Additionally, we found a higher incidence of BTX-A discontinuation among patients with acquired neurogenic bladder compared to those with congenital conditions: 11/17 (64%) vs. 13/38 (34%); *p* = 0.03. No statistically significant differences were found analyzing by sex (male 10/25 (40%) vs. female 14/30 (47%); *p* = 0.6) nor for urodynamic findings (OAB 15/29 (52%) vs. LCB 9/25 (36%); *p* = 0.2).

## 3. Discussion

The main goals of neurogenic bladder treatment are to preserve renal function, improve continence, and enhance quality of life as much as possible. As previously mentioned, CIC represents the primary approach associated with pharmacological therapy, which is using anticholinergic drugs to reduce detrusor overactivity while maintaining a high level of detrusor compliance and age-appropriate bladder capacity. However, this pharmacological therapy can cause significant side effects (e.g., dry mouth or eyes, constipation, intestinal disorders) or may not effectively control bladder pressure and detrusor overactivity. For these reasons, it may become necessary to consider invasive surgery, such as bladder augmentation. This surgery can lead to short and long-term complications, including the frequent formation of bladder stones, urinary infections, metabolic alterations, increased cancer risk, etc. A viable alternative, capable of avoiding or at least postponing this invasive surgery, particularly in pediatric patients, has emerged with the use of intradetrusor BTX-A injections [1,3,8,12,18].

BTX-A, a commercial form of Clostridium Botulinum Toxin-A, is a neurotoxin with a complex mechanism of action that affects various neurotransmitters and neuropeptides, influencing both sensory and motor nerves. Previously, BTX-A action was thought to be confined to the inhibition of acetylcholine presynaptic release. Today, it is understood that this neurotoxin also acts on calcitonin gene-related peptides on sensory receptors TRPV1 and P2X; BTX-A can reduce local inflammation and bladder pain. These effects at the bladder level can help preserve the UUT function, reduce bladder-filling pressure and detrusor overactivity, and improve bladder capacity and compliance. Nevertheless, to date, there is still limited data on the use of botulinum toxin in pediatric age. Naqvi et al. evaluated 30 pediatric patients (16 of them with OAB) in a 6-year study; these authors reported an increase in cystometric capacity and maximum neurogenic detrusor overactivity; they also found improved compliance in the LCB population after botulinum toxin injections. Hascoat et al. also noted a significant increase in bladder compliance after treatment with botulinum toxin in spina bifida patients in 6 of the 12 studies analyzed in their systematic review. Clinically, treatment with BTX-A has led to an improvement in incontinence episodes and vesicoureteral reflux. Their subsequent study on the effects of BTX-A in the pediatric neurogenic population revealed that only a third of the patients achieved full clinical and urodynamic effectiveness, but a 66% rate of resolution of incontinence was observed. Furthermore, a discrepancy emerged between the clinical and urodynamic results: more than half of the patients who showed clinical improvements still had pathological and potentially risky urodynamic parameters. This discrepancy can be explained, at least in part, by the effects of the neurotoxin on bladder afferent signaling, which may alter the sensory function of the urothelium and suburothelium, even without an effect on detrusor compliance and/or OAB. Figueroa et al. reported a series of 17 patients in a 4-year study, showing an improvement in symptoms (complete clinical resolution in 7 of 13 symptomatic patients) and urodynamic parameters (improvement in bladder capacity and compliance). Regarding our study, 7% (4/54 patients) discontinued treatment because it was no longer necessary (due to the resolution of NLUTD after neurosurgical treatment). At the last available follow-up, excluding these 4 patients, 34% (17/50 patients) showed a reduction of BTX-A efficacy, while 66% (33/50 patients) exhibited long-term efficacy. Moreover, it is known that BTX-A injection at the urethral sphincter can effectively decrease urethral resistance and improve voiding. Mosiello et al. evaluated the efficacy of combined treatment with suburethral injection of dextranomer/hyaluronic acid and intratradetrusor BTX-A injections for the treatment of VUR in patients with neurogenic bladder not responsive to CIC and anticholinergic drugs, with good results. Therefore, BTX-A can be combined with other minimally invasive treatments for continence. BTX-A treatment can also improve bowel dysfunction, commonly present in SB patients. The mechanism of action on bowel function is not well understood; it might be related to the shared innervation or the absorption of the toxin by the structures adjacent to the bladder. BTX-A injections are usually well tolerated by patients, although the level of satisfaction with the procedure is controversial [1,2,8,9,12,18,19,20,21,22,23,24].

Side effects can vary. The most common are post-injection UTIs, reported in 20.4% of cases within 3 months after the procedure and in 33% during the 6 months postoperative. Urinary retention is also quite frequent, necessitating catheterization in 5.4–14.6% of patients. Hematuria (occurring during or soon after the treatment) has an incidence of 3.6–9%; more modern, less traumatic needles may contribute to a further reduction in peri-procedural bleeding. To decrease the incidence of side effects, a specific protocol has been implemented at our hospital, including the positioning of a bladder catheter for 24 h after the injections to facilitate continuous emptying of the bladder and to prevent bladder over-distension, which increases the risk of hematuria, or the need to perform CIC soon after the procedure. Rarer complications include muscle weakness or hyposthenia and paralysis of distant muscles. Increasing post-void residual, acute urinary retention, and general weakness are less common in children than in adults. Side effects are considered transient (lasting approximately 2 weeks–2 months). Some of these effects are believed to be dose-dependent, such as weakness and hyposthenia. Regarding the dosage of BTX-A, there is no consensus on the appropriate dose of BTX-A for treatment in pediatric patients. The most recent multicentric international study recommends a dosage of 6 U/kg (maximum 200 U). We use a dosage of 8 U/kg (maximum 200 U) [1,3,25,26].

There is also no unanimous consensus on the definition of “failure” of BTX-A therapy. Peyronnet et al., in a survey of adult patients with neurogenic bladder and OAB, highlighted that the main criteria for therapeutic failure include a lack of clinical (incontinence) and urodynamic (maximum detrusor pressure) improvement. In the case of failure of the first BTX-A injection, most experts still administer a second treatment with BTX-A at a higher dose. Mailho et al. also analyzed the definition of failure in BTX-A therapy for neurogenic bladder. They gathered the opinion of 16 experts, reaching an initial consensus on the following parameters: persistence of OAB with maximum detrusor pressure > 40 cmH_2_O and/or alterations of compliance and/or persistent incontinence and/or persistent urgency and/or CIC > 8/day and/or duration of therapy effects < 3 months. Over the years, various factors have been suggested as predisposing elements to the failure of BTX-A therapy. Among these, the presence of a low-compliance bladder (likely due to increased extracellular matrix and resulting fibrosis, which reduces the diffusion of the toxin inside the detrusor) and urethral sphincter deficiency have emerged, often found in patients with spina bifida (whether associated with low-compliance bladder or OAB) [23,27,28].

A limitation of BTX-A therapy is its temporary effect, which is time-limited and varies over time and from patient to patient, with peak effectiveness occurring 4–6 weeks after the procedure but lasting approximately 3–12 months. Consequently, repeated administrations of the toxin are necessary [3,15]. We repeated BTX-A treatment for an average of 16 months (6–53 months), with an average of 6.4 treatments for patients during the study period.

Treatment with BTX-A is burdened by a significant limitation: the abandonment of therapy. It is reported that approximately 39.6–49.1% of adult patients discontinued treatment over time, with more than 50% stopping within 10 years from the first injection. We found a higher discontinuation rate of 76% during our average follow-up of 10.2 years, particularly among patients with acquired neurogenic bladder. The reasons behind this high rate of abandonment have been extensively studied, primarily within the adult population. The necessity to repeat the treatment, coupled with the requirement of general anesthesia or sedation in most pediatric cases, may be one of the primary reasons for discontinuation of the treatment, decided by patients and/or their parents. Furthermore, some studies have highlighted potential neurotoxicity related to anesthesia in children, which must, however, be weighed against the possible long-term effects of anticholinergic drugs on the central nervous system. In their series (including patients with neurological or idiopathic OAB), Mohee et al. reported an abandonment rate of 61.3% at 3 years from the start of therapy and 63.8% after 5 years; the main reasons cited involved tolerability issues (e.g., related to post-procedural complication, need for CIC), while loss of efficacy was considered less important. Other case studies, such as that reported by Baron et al., have instead highlighted the lack of urodynamic or clinical effectiveness as the main cause of discontinuation (43.7%); other reasons included the patient’s decision (28.1%), worsening of incontinence (14.1%), progression of neurological problems related to the underlying condition. Factors predisposing to the abandonment of BTX-A therapy include the presence of urinary incontinence before the start of therapy (compared to continent patients), an acontractile bladder with low compliance, and a very small bladder capacity; the presence of an open bladder neck is considered an independent predictor of poor response after BTX-A injections. The lack of compliance has been identified as another critical factor in the long-term abandonment of therapy; indeed, many patients prefer to return to pharmacological therapy, even with less control of symptoms, and avoid repeated treatments with BTX-A. In the adult population, conducting this therapy in an outpatient setting with local anesthesia, for example, can improve compliance. However, the same cannot be said for the pediatric population, where the procedure is performed in the operating room and with general anesthesia during brief hospitalization, as previously mentioned [2,14,16,17,18,29].

The necessity to perform multiple intradetrusor injections and the need to repeat the treatment over time have raised concerns about the possible progression of bladder fibrosis, a common finding in neurogenic bladder, which could limit the diffusion of the toxin into the bladder wall and thus reduce the long-term effectiveness of therapy. Several studies involving both humans and rats have not demonstrated an increase in bladder fibrosis following treatment with BTX-A. Indeed, some studies have shown histological improvement (as in the early treatment of spinal cord injuries). Even in the pediatric population, no significant histological changes were observed following repeated treatments in long-term follow-up, as we reported in a previous study involving 36 patients who underwent at least 5 treatments. Another hypothesis for the reduction in the clinical response to BTX-A over time was the development of neutralizing antibodies. The presence of these specific antibodies is well documented in patients treated for cervical dystonia or spasticity, but their formation and clinical significance following bladder injection are still unclear. Moreover, some authors have highlighted a discrepancy between patients considered “non-responders” to BTX-A and the presence of antibodies: 40% of the “responders” patients had anti-toxin antibodies, 50% of the “non-responders” did not have these antibodies [3,12,14,30,31,32,33,34,35,36]. Additional potential reasons for discontinuing BTX-A therapy, particularly in the fragile population represented by adolescents and young adults, include poor symptom resolution (e.g., incontinence, UTIs), the desire for a “definitive solution” (e.g., major surgery), or resistance to their underlying chronic condition. We observed a higher rate of discontinuation among patients with acquired neurogenic bladder, possibly due to a refusal to accept the new pathological condition. It is known that adolescents generally have a worse health profile compared to their peers aged 20–30 years, especially if they suffer from neurological conditions. In the case of neurogenic bladder, patients often resist first-line behavioral recommendations and pharmacological treatments. The transition from a pediatric urology department to an adult urology center, which usually occurs at 18 years of age, is particularly challenging for patients who have undergone prolonged treatment in their childhood/adolescence. Indeed, it emerged that only 50% of adolescent and young adult patients were adequately prepared and educated for the transition of care. Among the main issues identified were poor organization of the transition, intimidation of the new environment with a sense of loss of familiar references, and poor independence of the patient [13].

Limits of the study: a single-center, retrospective study. The retrospective nature of a single-center study certainly weakens its statistical power, as we were not able to use the STROBE guidelines, given that it is an observational study. No assessment of quality of life was conducted. No questionnaire about patients/parents’ decision to abandon BTX-A therapy was administered.

## 4. Conclusions

BTX-A treatment in children, as in adults, appears to be safe and effective in neurogenic bladder that is refractory to the first-line therapy. However, even in pediatric patients, there is a progressive loss of adherence to this therapy, which is higher than that reported in adult case series. BTX-A should not be considered a definitive therapy due to its possible reduction in efficacy over time.

Further studies are needed to clarify the factors contributing to this low adherence to BTX-A therapy in the pediatric and adolescent populations. These studies should aim to better define this high abandonment rate and develop strategies to prevent it. A multicenter study could be very useful for clarifying the situation of BTX-A in the pediatric field, as current literature on this subject is limited. This work is generated within the European Reference Network for Rare Urogenital Diseases and Complex Conditions (ERN EUROGEN).

## 5. Materials and Methods

### 5.1. Ethical Aspect

The study was approved by the Ethical Committee of our hospital (number 200602R001820). Written informed consent was obtained from all subjects involved in the study (patients/parents/caregivers).

### 5.2. Inclusion Criteria

Charts of all patients affected by neurogenic bladder and treated in our division between December 1997 and December 2022 with Botulinum Toxin-A intradetrusor injection were retrospectively reviewed.

We collected all demographic and clinical data from patients’ charts.

Inclusion criteria were: patients with neurogenic bladder refractory to first-line treatment who underwent first BTX-A injection at least 10 years before the end of the study (before December 2012), with ≥3 BTX-A treatment performed during the study period.

We arbitrarily considered the repetition of at least 3 BTX-A retreatment as “clinical responders” and “long-term therapy”.

### 5.3. Exclusion Criteria

We excluded all patients with incomplete medical records, first BTX-A injection performed < 10 years from study end, <3 BTX-A retreatments.

### 5.4. Patients’ Evaluation

All patients with neurogenic bladder dysfunction were initially evaluated in our outpatient clinic, with clinical evaluation of signs and symptoms, laboratory test (blood and urine exams), urinary ultrasound or other radiological images if needed (e.g., voiding cystourethrography, magnetic resonance), and urodynamic/videourodynamic evaluations, as recommended by the International Children’s Continence Society (ICCS) [37]. We propose BTX-A injection as a second-line treatment in patients with neurogenic bladder that is not responding (clinically, radiologically, and urodinamically) to first-line treatments (anticholinergic and CIC).

### 5.5. BTX-A Treatment Protocol

From 2003, we standardized our BTX-A injection protocol as previously reported [3,38]. During a short hospitalization, under general anesthesia in the operating room, 8 U/kg (maximum 200 U) of Onabotulinum Toxin-A (Botox, 100 U Allergan, AbbVie S.r.l., Campoverde di Aprilia, Latina, Italy) were injected into the detrusor (multiple sites injections), sparing the trigone, using a rigid cystoscope and a flexible needle. Each injection was 1 mL (10 U/1 mL of saline solution). All patients underwent an endoscopic cold-cup biopsy immediately before the injection to analyze eventual histological modification. A bladder catheter (Foley catheter) was left for 24 h post-procedure.

### 5.6. Follow-Up

All patients were enrolled in our follow-up. Each patient was evaluated at least once a year. Response on BTX-A therapy was evaluated based on patients’ satisfaction of symptom control and continence status based on diaries (dryness through CIC, number of CIC per day, leaking episodes); presence of urinary tract infections; laboratory tests (blood and urine); upper urinary tract ultrasound alteration/worsening/improving. Other tests were performed based on specific findings: invasive urodynamic (or video-urodynamic) exam, non-invasive urodynamic (uroflowmetry with post-void residue evaluation) [39] if possible due to underlying pathology, and voiding cystourethrography.

We then compared these data with those obtained before the BTX-A injections to evaluate the efficacy of the treatment.

We divided patients into four categories based on current treatment at the last available follow-up:

- continuing BTX-A treatment
- not continuing BTX-A treatment
- lost at follow-up (including deceased patients)
- BTX-A no longer necessary (due to underlying condition improvement).

In the case of discontinuation of BTX-A treatment, cause and eventually alternative treatments were recorded.

We considered all patients who did not show up at the following scheduled examination and/or treatment and did not contact us in the following year to rearrange it to be lost at follow-up.

### 5.7. Statistics

Data were analyzed using MedCalc^®^ Statistical Software version 20.2016 (MedCalc Software Ltd., Ostend, Belgium: https://www.medcalc.org, (accessed on 19 March 2024)). Results are reported as numbers with percentages or mean, specifying SD and range; *p* < 0.05 was considered statistically significant.

## Figures and Tables

**Table 1:** Causes of neurogenic bladder.

Cause of Neurogenic Bladder:	N of pts (%)
Congenital:	37 (69%)
Myelomeningocele	19
OSD	11
Caudal regression	3
Chiari malformation 1	2
CDG	1
Tuberosus sclerosis	1
Acquired:	17 (31%)
SCI	3
Sacrococcygeal teratoma	1
Neuroblastoma	3
Iatrogenic injury *	3
Arachnoid cyst	3
Cerebral palsy	4

N of pts: number of patients, OSD = occult spinal dysraphism, CDG = congenital disorders of glycosylation, SCI = spinal cord injury. * Iatrogenic injury: medullary ischemia post-cardiothoracic surgery (2 patients), chemotherapy (1 patient).

## Data Availability

Patients’ data are reported in this paper. Other data are available in their clinical charts if necessary.

## List of Abbreviations

BTX-A	Onabotulinum Toxin-A
NLUTD	Neurogenic Lower Urinary Tract Dysfunction
SB	Spina Bifida
CIC	Clean Intermittent Catheterization
OSD	occult spinal dysraphism
UUT	upper urinary tract
UTI	urinary tract infections
VUR	vesicoureteral reflux
OAB	overactive bladder
LCB	low-compliance bladder
CDG	congenital disorders of glycosylation
SCI	spinal cord injury
FU	follow-up
SNM	sacral neuromodulation
ICCS	International Children’s Continence Society
